# A century of exercise physiology: key concepts in regulation of glycogen metabolism in skeletal muscle

**DOI:** 10.1007/s00421-022-04935-1

**Published:** 2022-03-30

**Authors:** Abram Katz

**Affiliations:** Åstrand Laboratory, Department of Physiology, Nutrition and Biomechanics, The Swedish School of Sport and Health Sciences, GIH, Stockholm, Sweden

**Keywords:** Glycogen, Muscle, Phosphorylase, Glycogen synthase, Glycogenin, Exercise

## Abstract

Glycogen is a branched, glucose polymer and the storage form of glucose in cells. Glycogen has traditionally been viewed as a key substrate for muscle ATP production during conditions of high energy demand and considered to be limiting for work capacity and force generation under defined conditions. Glycogenolysis is catalyzed by phosphorylase, while glycogenesis is catalyzed by glycogen synthase. For many years, it was believed that a primer was required for de novo glycogen synthesis and the protein considered responsible for this process was ultimately discovered and named glycogenin. However, the subsequent observation of glycogen storage in the absence of functional glycogenin raises questions about the true role of the protein. In resting muscle, phosphorylase is generally considered to be present in two forms: non-phosphorylated and inactive (phosphorylase **b**) and phosphorylated and constitutively active (phosphorylase **a**). Initially, it was believed that activation of phosphorylase during intense muscle contraction was primarily accounted for by phosphorylation of phosphorylase **b** (activated by increases in AMP) to **a**, and that glycogen synthesis during recovery from exercise occurred solely through mechanisms controlled by glucose transport and glycogen synthase. However, it now appears that these views require modifications. Moreover, the traditional roles of glycogen in muscle function have been extended in recent years and in some instances, the original concepts have undergone revision. Thus, despite the extensive amount of knowledge accrued during the past 100 years, several critical questions remain regarding the regulation of glycogen metabolism and its role in living muscle.

## Introduction

The earliest evidence for an important role of carbohydrate in muscle function is likely the observation of an enhanced glucose uptake in equine masseter muscle during chewing (Chauveau and Kaufmann 1887). Subsequent studies, almost a century ago, reinforced the importance of this observation in exercising humans (Christensen and Hansen 1939; Levine et al. 1924). Although it was known that glycogen was degraded during muscle contraction (Cori and Cori 1933; Cori 1956), the relative contributions of extracellular glucose and intramuscular glycogen to muscle carbohydrate utilization (glycolysis) during exercise were unknown. About 60 years ago, infusion of a trace amount of ^14^C-glucose into a subject prior to onset of moderate exercise resulted in the conclusion that muscle glycogen also contributed to muscle energy turnover, especially during the initial phase of exercise (Reichard et al. 1961). However, no quantitative estimates of the contribution of glycogen degradation to carbohydrate utilization were obtained. Shortly after, the pioneering studies of Bergström and Hultman demonstrated that muscle glycogen was the major carbohydrate substrate during exercise in humans (Bergstrom and Hultman 1966a, 1967). Later studies provided quantitative measurements of glucose utilization (arteriovenous differences x blood flow) and glycogen degradation during prolonged moderate exercise, allowing for the conclusion that glycogen degradation accounted for > 80% of muscle glycolysis throughout exercise, and the contribution of glycogen breakdown to glycolysis increased as a function of exercise intensity (Katz et al. 1986a, 1991b). Today, it is well established that glycogen is the major carbohydrate source for muscle energy turnover during many if not most forms of physical exercise (Hargreaves and Spriet 2020).

Glycogen is a branched, glucose polymer whose discovery in 1857 is credited to Claude Bernard (cited in (Young 1957)). Almost 100 years later, the enzyme responsible for glycogen degradation, glycogen phosphorylase, was discovered. (Cori et al. 1939; Green and Cori 1943). The characterization of phosphorylase and its central role in glycogen metabolism led to the award of the 1947 Noble prize. Subsequent studies of phosphorylase and glycogen metabolism led to additional Noble prizes in 1970 (sugar nucleotides), 1971 (cAMP) and 1992 (protein phosphorylation) (Roach et al. 2012). While small amounts of glycogen are found in many mammalian cell types (including astrocytes, adipocytes and renal cells), the concentrations are highest in liver, skeletal and cardiac muscle. Noteworthy, is that skeletal muscle stores ~ 80% of total body glycogen. There have been several reviews in recent years that have summarized different aspects of glycogen metabolism, including pathologies (Katz and Westerblad 2014; Roach et al. 2012; Vigh-Larsen et al. 2021; Gentry et al. 2018; Almodovar-Paya et al. 2020). In the present review, an emphasis is placed on recent views regarding the regulation of glycogen metabolism during repeated contractions/exercise and recovery, as well as the role of glycogen in force generation and fatigue in living muscle.

## Enzymes of glycogen metabolism

I will begin with a brief description and characterization of the key enzymes of glycogen metabolism in the purified state or in dilute extracts, as this will serve as a comparative basis for the discussion in living muscle (see below). The reader is referred elsewhere for more detailed biochemical/molecular/structural characterization of the enzymes (Roach et al. 2012; Johnson 1992; Sprang et al. 1991; Newgard et al. 1989).

### Phosphorylase

Phosphorylase, together with debranching enzyme, catalyze the breakdown of glycogen (Glycogen_n_ + P_i_ → Glycogen_n-1_ + glucose 1-P). Phosphorylase is rate-limiting for glycogenolysis (Newsholme and Leech 1985) and the reaction is considered to be irreversible in the cell. Phosphorylase exists in two forms: phosphorylated (phosphorylase **a,** generally considered to be constitutively active) and non-phosphorylated (phosphorylase **b,** generally considered to be inactive in muscle at rest). Both forms of the enzyme are subject to allosteric regulation by various ligands. AMP primarily activates phosphorylase **b**, but can also activate phosphorylase **a** depending on the assay conditions (Lowry et al. 1964; Johnson 1992). Glucose 6-P and ATP inhibit phosphorylase **b** but have little effect on phosphorylase **a** (Morgan and Parmeggiani 1964b). Phosphorylase is also subject to regulation by substrate availability (glycogen and P_i_) (Chasiotis 1983). The K_m_ values of both forms of phosphorylase for glycogen in vitro are extremely low (≤ 3 mmol glucosyl units/l) (Brown and Cori 1961; Lowry et al. 1964; Morgan and Parmeggiani 1964b). The K_m_ values for P_i_ are low for phosphorylase **a** (~ 5 mM) and higher for phosphorylase **b** (~ 25 mM) (Chasiotis 1983; Kasvinsky and Meyer 1977; Morgan and Parmeggiani 1964a; Ren and Hultman 1990). Importantly, increases in AMP lower the K_m_ values for P_i_ for both forms of the enzyme to similar values (Morgan and Parmeggiani 1964b, 1964a). Finally, phosphorylase is subject to covalent modification by phosphorylation-dephosphorylation reactions that are catalyzed, respectively, by phosphorylase **b** kinase and protein phosphatase-1 and -2A (Fischer 2013; Johnson 1992; Roach 2002; Ingebritsen et al. 1983). Phosphorylation of Ser-14 results in conversion of phosphorylase **b** to **a** (Johnson 1992). More recently, other forms of covalent modification have been described: acetylation, oxidation and nitration, all of which result in inhibition of phosphorylase activity (Zhang et al. 2012; Dairou et al. 2007; Mathieu et al. 2016).

### Glycogen synthase

Glycogen synthase (GS), together with branching enzyme, catalyze the synthesis of glycogen (Glycogen_n_ + UDP-glucose → Glycogen_n+1_ + UDP). The limiting factor for glycogen biogenesis in living cells is generally considered to reside at either the glucose transport step or GS activity, depending on the experimental conditions, as discussed elsewhere (Roach et al. 2012). The initial evidence for the existence of GS was described in liver extracts (Leloir and Cardini 1957). Subsequently, purification and characterization of the enzyme in skeletal muscle was performed by Larner and colleagues (Larner and Villar-Palasi 1971). As is the case for phosphorylase, GS is also controlled by substrate availability, allosteric regulation by various ligands (especially glucose 6-P) and covalent modification (Larner and Villar-Palasi 1971; Roach et al. 2012). The K_m_ of GS for glycogen is in the low uM range (about 1–20 µmol glucosyl units/l) (Larner et al. 1976; Brown et al. 1965). The K_m_ of essentially non-phosphorylated GS (GS-I, indicating independence of glucose 6-P) for UDP-glucose is on the order of 1 mM in the absence of glucose 6-P and about 0.05 mM in the presence of glucose 6-P, while the K_m_ of highly phosphorylated GS (GSD, indicating dependence on glucose 6-P) for UDP-glucose is > 1 M in the absence of glucose 6-P and about 0.2 mM in the presence of glucose 6-P (Roach et al. 1976). Both forms of GS are subject to inhibition by nucleotides and P_i_, with GSD being more sensitive to such inhibition, especially at physiological concentrations of glucose 6-P (Larner and Villar-Palasi 1971). Glucose 6-P decreases the K_m_ of GS-I for UDP-glucose and increases the maximal velocity (V_max_) of GSD (Larner and Villar-Palasi 1971). The phosphorylation of GS is catalyzed by a number of protein kinases (Roach et al. 2012), whereas dephosphorylation is catalyzed by protein phosphatase-1 and -2A (Alemany et al. 1984). However, in contrast to phosphorylase, GS contains nine phosphorylation sites, which results in a complex regulation. Studies of these sites led to the concept of hierarchal phosphorylation, where introduction of one phosphate enables the addition of a second (Roach et al. 2012). More recently described covalent modifications of GS include O-linked attachment of N-acetylglucosamine as well as acetylation. The extent to which these modifications alter skeletal muscle GS activity under physiological conditions remains to be established (Roach et al. 2012).

### Glycogenin

The mechanism for de novo glycogen biogenesis was a mystery for many years. Salsas and Larner demonstrated that glucose could act as an acceptor from the glucosyl donor UDP-glucose in the presence of GS to form maltose (Salsas and Larner 1975). However, the K_m_ of GS for glucose was 900 mM which appeared to preclude an in vivo role for this reaction. It had long been reported that isolated glycogen was always associated with protein, and this protein was subsequently identified as glycogenin (Kennedy et al. 1985). Glycogenin is a self-glucosylating protein that transfers glucose from UDP-glucose to a tyrosine residue (tyr-194). The self-glucosylation continues until about ten glucose residues are incorporated, whereafter GS and branching enzyme continue to form mature glycogen (Lomako et al. 2004; Smythe and Cohen 1991). While the naturally occurring glucosyl donor for glycogenin is UDP-glucose, other purine nucleotides are not utilized, whereas two pyrimidine nucleotides (CDP-glucose and TDP-glucose) are functional (Alonso et al. 1995a). Glycogenin has an absolute requirement for Mn^2+^, while ATP, UTP, UDP and especially CDP inhibit activity (Alonso et al. 1995b, 1995a; Cao et al. 1993; Manzella et al. 1994; Roden et al. 1994).

## Regulation of glycogenolysis in living muscle during contraction/exercise

### Phosphorylation of phosphorylase b

Skeletal muscle is unique in its ability to accelerate energy turnover several-100- fold in less than a second (Hultman and Sjoholm 1983b; Sahlin et al. 1998). Accordingly, the increased rate of ATP utilization must be met by an equivalent rate of ATP production to maintain force generation. Such high energy turnover rates can be maintained for a few seconds at most. Under such conditions, the energy requirements are met almost exclusively by anaerobically derived ATP from phosphocreatine (PCr) and glycogen degradation. It follows that there must be a rapid activation of phosphorylase for the latter to occur. Early research suggested that covalent phosphorylation of phosphorylase **b**, resulting in formation of phosphorylase **a**, was in large responsible for activation of glycogenolysis during muscle contractions (Cori 1956; Danforth et al. 1962). Formation of phosphorylase **a** was rapid and could be detected in less than 1 s (Danforth and Lyon 1964). Indeed, it was estimated that during intense isometric contractions of isolated frog muscle at 30 °C, activation of phosphorylase occurs with a half time of 0.7 s (Danforth et al. 1962). Similarly, high-frequency electrical stimulation of isolated fast-twitch mammalian muscle increases the fraction of phosphorylase **a** from ~ 10% at rest to ~ 70% within 1 s (Blackwood and Katz 2019). If high-frequency stimulation continues for more than 10–20 s, phosphorylase **a** activity declines and can even reach values below baseline, while significant degrees of glycogenolysis are maintained in rodent muscle (Piras and Staneloni 1969; Conlee et al. 1979; Rahim et al. 1980). Similar findings were subsequently demonstrated in human skeletal muscle during both isometric (anaerobic) and dynamic (aerobic) exercise (Chasiotis et al. 1982).

Early on, it was noted that in a strain of mice lacking phosphorylase **b** kinase, there was no formation of phosphorylase **a** during repeated contractions. And although these mice exhibited a blunting of glycogenolysis during the initial seconds of muscle contractions induced by electrical stimulation, subsequently, there was clear glycogen breakdown (Danforth and Lyon 1964). In a later study, glycogenolysis was actually increased in muscle deficient in phosphorylase **b** kinase during muscle contractions induced by electrical stimulation (Rahim et al. 1980). The increased rate of glycogenolysis in phosphorylase **b** kinase-deficient muscle during the initial 30 s of electrical stimulation was associated with a sixfold higher rate of inosine 5’-monophosphate (IMP) formation (Rahim et al. 1980). Phosphorylase **b** kinase deficiency in human skeletal muscle (Glycogen Storage Disease, GSD IX) is exceedingly rare. Moreover, it is not always defined by complete loss of kinase activity and can be associated with elevated levels of glycogen phosphorylase (Andersen et al. 2020), which complicates use of this model to study regulation of glycogenolysis. Taken together, the data question the conclusion that conversion of phosphorylase **b** to **a** is the major mechanism for activation of glycogenolysis during intense muscle contraction. This view is further supported by numerous studies that have used adrenaline to increase formation of phosphorylase **a** either in muscles at rest or during contraction and found that the increases in the rate of glycogenolysis are negligible as compared to those observed during muscle contraction in the absence of adrenaline (reviewed in (Katz and Westerblad 2014)).

### Inorganic phosphate

An alternative explanation for the regulation of glycogenolysis during increased energy turnover was suggested to be a limitation of the substrate P_i_, which increases when PCr is degraded during exercise (Chasiotis 1983); indeed, such a possibility was discussed more than 60 years ago (Cori 1956). It was shown that in the resting state and during adrenaline infusion that resulted in marked conversion of phosphorylase **b** to **a**, glycogenolysis was nominal and this was attributed to low P_i_ levels at the enzymatic site. Increasing P_i_ during circulatory occlusion (owing to PCr breakdown), which was associated with a decrease in phosphorylase **a** levels, resulted in an activation of glycogenolysis, supporting a physiological role for P_i_ availability (Chasiotis and Hultman 1983). In other studies, however, circulatory occlusion resulted in an increase in phosphorylase **a** levels (Katz 1997). Regardless of the changes in phosphorylase **a**, is the finding that the rate of glycogen degradation is very low during circulatory occlusion (< 1 mmol/min/kg dry muscle). Another approach to assess the role of P_i_ in control of glycogenolysis is to first elevate P_i_ levels with short-term intermittent contractions and then induce ischemia and measure the rate of glycogenolysis during the ischemic period (during which elevated levels of P_i_ are maintained). Such experiments show negligible glycogenolysis during the ischemic period (Ren and Hultman 1990). Even when adrenaline infusion was performed during the intermittent contractions, resulting in > 90% of phosphorylase being phosphorylated (in **a** form) at the onset of ischemia, together with elevated P_i_ levels, glycogenolysis was still not detectable during the following ischemic period. The role of P_i_ levels during contraction was also examined in isolated fast-twitch mouse muscle that lacked creatine kinase (CK) activity, and therefore, cannot break down PCr (Katz et al. 2003). Short-term repeated contractions of CK-deficient muscle did not affect PCr levels and the increase in P_i_ content was minimal (~ 35% vs. almost 500% in wild-type control muscle). Fractional activity of phosphorylase (fraction of **a**/(**a** + **b**)) was about 10% in both groups in the basal state and increased to ~ 50% and 40% following 20 s of repeated contractions in control and CK deficient muscle, respectively. Despite the lower levels of P_i_ and lower phosphorylase **a** activity in CK-deficient muscle, glycogenolysis was actually increased (Katz et al. 2003). In summary, changes in P_i_ content, either with or without increases in phosphorylase **a** activity cannot account for the activation of phosphorylase during high rates of energy turnover in living muscle.

### Glycogen

During intense short-term exercise, the rate of glycogen degradation is fairly constant (Sahlin et al. 1975; Hultman and Sjoholm 1983a). During prolonged moderate exercise, the rate of glycogenolysis is initially high but decreases progressively (Sahlin et al. 1990b). The rate of glycogenolysis increases with exercise intensity in an exponential manner (Saltin and Karlsson 1971). As discussed above, the K_m_ for glycogen is likely to be less than 3 mmol glucosyl units/l or < 10 mmol glucosyl units/kg dry muscle (to convert from mM to dry wt. multiply by 3.3). This can be compared with a normal resting glycogen content in humans of about 400 mmol glucosyl units/kg dry muscle, which would suggest that glycogen is rarely limiting for glycogenolysis. However, this assumes that the K_m_ in vitro also applies to in vivo conditions. This can be questioned considering that glycogen exists as particles of unequal size rather than as a homogeneous solution (Richter and Galbo 1986; Sjostrom et al. 1982) and depletion of glycogen particles can be restricted to specific cellular loci (Ortenblad et al. 2013). In this context, it is noteworthy that when relatively high-intensity exercise is performed, glycogen degradation rates are similar despite large differences in initial glycogen content (Ren et al. 1990; Sahlin et al. 1989; Spencer and Katz 1991; Spriet et al. 1990). However, at values approaching 10 mmol glucosyl units/kg dry muscle, there is clearly an attenuation of hexose phosphate accumulation, although glycolysis is maintained (Hultman and Sjoholm 1983b). These findings are consistent with the idea that glycogen levels do not limit glycogenolysis unless very low levels are reached during conditions of high energy turnover. In contrast, during low/moderate-intensity exercise, a higher initial glycogen content is associated with a higher rate of glycogenolysis (Galbo et al. 1979; Hespel and Richter 1992; Richter and Galbo 1986; Spencer et al. 1992). The reason for these differential responses to glycogen levels between high vs. low/moderate exercise intensity is not clear. One factor that may be considered is that the low/moderate-intensity exercise is normally of a dynamic nature (e.g., leg cycling) with an intact circulation. This raises the possibility that phosphorylase could be affected by circulatory factors (e.g., hormones or substrates). Indeed, an early study demonstrated that infusion of glucose during submaximal exercise attenuated muscle glycogen utilization (Bergstrom and Hultman 1967). Moreover, often, the protocols used to induce marked differences in initial glycogen levels (e.g., glycogen depleting exercise followed by altered diet) result in differences in hormone and substrate levels in blood at the onset of exercise with high vs. low glycogen (Galbo et al. 1979; Spencer et al. 1992). However, it has been demonstrated that prior glycogen depletion of one leg followed by submaximal two-legged cycling the next day (low vs. high glycogen leg) still results in greater glycogen degradation in the high glycogen leg, although both legs are exposed to the same extracellular milieu (Gollnick et al. 1981). On the other hand, it is well established that exercise training results in an increase in the basal glycogen level, but a decrease in the rate of glycogenolysis during submaximal exercise (Chesley et al. 1996; Phillips et al. 1996). In summary, it appears that glycogen levels are not limiting for phosphorylase activity in living muscle during high rates of energy turnover, unless extremely low levels are reached. However, during low/moderate exercise intensities, this question remains unresolved.

### AMP

As described above, during high rates of energy turnover, the initial transformation of phosphorylase **b** to **a** is reversed as contraction continues and phosphorylase **a** values can even decrease below basal. This occurs when force is decreasing or when force or workload is constant (Chasiotis et al. 1982; Conlee et al. 1979; Danforth and Helmreich 1964; Piras and Staneloni 1969; Aragon et al. 1980b). The decrease below baseline is even observed in human muscle following prolonged submaximal exercise to fatigue (Jiao et al. 1999). Despite the reversal of phosphorylase **a**, glycogen breakdown continues throughout exercise, although the rate decreases with time (Sahlin et al. 1990b). To explain high rates of glycogenolysis in the essential absence of phosphorylase **a,** activation of phosphorylase **b** must occur. The most potent physiologic activator of phosphorylase **b** is AMP although IMP can also activate, albeit the enzyme is far less sensitive to IMP than to AMP (Aragon et al. 1980b). Depending on assay conditions and tissue preparations, the K_a_ of phosphorylase **a** for AMP has been reported to range from 0.5 to 5 µM and of phosphorylase **b** from 30 to 100 µM (Aragon et al. 1980b; Lowry et al. 1964; Morgan and Parmeggiani 1964b; Katz et al. 2003; Cuenda et al. 1995). Total tissue AMP concentrations in skeletal muscle generally range from ~ 50 to 200 µM and do not change much with exercise/contractions (Katz et al. 2003; Aragon et al. 1980b; Ren et al. 1988; Conlee et al. 1979). If these K_a_ values, derived from purified enzyme preparations or dilute crude extracts, and total tissue AMP values are relevant, then phosphorylase should be strongly activated already in the resting state. Clearly, that is not the case. It is generally believed that the free concentration of AMP is much lower than the total tissue content owing to protein binding or sequestration in the cell (reviewed in (Sahlin 1991)). Calculations of free AMP, using total tissue values of ATP, PCr and creatine and assuming that the creatine kinase and adenylate kinase reactions are at equilibrium at pH 7.0, yield free AMP values at rest that are about 0.2 µM and following intense repeated contractions of almost 70 µM (Aragon et al. 1980b). At pH 6.6 (representative of muscle pH after intense exercise (Sahlin et al. 1975)), the corresponding values are 0.04 and 13 µM, respectively. Aragon et al. suggested that these AMP values would not be sufficient to account for the measured rates of glycogenolysis during electrical stimulation of rat skeletal muscle in situ (Aragon et al. 1980b). However, the large increases in IMP (> 1 mM) would be sufficient to account for the high glycogenolytic rates (Aragon et al. 1980b). It is questionable whether either of these estimates/measurements yield relevant values concerning the regulation of phosphorylase and glycogenolysis. First, there are questions regarding the assumptions involved in calculations of free ADP and AMP (Sahlin 1991). Second, experimental findings speak against the validity of such calculations and conclusions as illustrated by the following. By performing intense repeated contractions/exercise, one can reach essential depletion of PCr and marked decreases in ATP that correspond to stoichiometric increases in IMP. Under such conditions, high values of calculated free AMP and measured IMP are obtained, and coincide with high rates of glycogenolysis/glycolysis (Sahlin et al. 1990a; Katz and Raz 1995). However, when similar changes in calculated free AMP and measured IMP are achieved during prolonged ischemia or during recovery from intense exercise under ischemic conditions, the rate of glycogenolysis is negligible (Sahlin 1991; Sahlin et al. 1990a; Katz and Raz 1995). Such data indicate that the calculated free AMP values and/or the K_a_ values for AMP do not represent those necessary to account for activation of phosphorylase in living muscle.

From the above discussion, it was concluded that high rates of glycogenolysis are closely linked to factors associated with the contraction processes (Sahlin 1991). One obvious factor would be Ca^2+^, which is released by sarcoplasmic reticulum to initiate cross-bridge binding, breakdown of ATP and force generation/cross-bridge cycling. While it has long been recognized that Ca^2+^ activates phosphorylase **b** kinase (Cohen 1982), there is no evidence that Ca^2+^ can activate phosphorylase **b** directly. This, however, does not rule out the possibility that Ca^2+^ functions through another mechanism that results in activation of phosphorylase **b**, independent of phosphorylase **b** kinase. Such a mechanism, if it exists, remains to be demonstrated. Noteworthy is the observation that the inverse relationship between PCr and lactate (which indicates a link between energy metabolism and glycogenolysis/glycolysis) is maintained both in the presence and absence of Ca^2+^ transients (Ortenblad et al. 2009), which does not support a key role for Ca^2+^ in activation of phosphorylase during intense contraction/exercise. Concomitant with increases in Ca^2+^ during such conditions, the breakdown of ATP will lead to increases in ADP and AMP. However, owing to temporal and spatial gradients the “transient” changes in ADP and AMP during contraction are either too small or too rapid to be detected with current techniques (Sahlin 1991). Thus, the relevant species of AMP, i.e., the free concentration at the enzymatic site during contraction (Katz and Westerblad 2014) have yet to be demonstrated.

When AMP does reach sufficiently high levels, in addition to activating phosphorylase, as well as phosphofructokinase (i.e., glycolysis), it is deaminated by AMP deaminase to IMP and NH_3_. The removal of the latter products is very slow and, therefore, they have been used as surrogates to reflect the relevant (transient) increases in AMP (Katz et al. 1986b; Sahlin 1991; Katz and Westerblad 2014; Katz and Sahlin 1990). While this explanation may appear to be a reasonable alternative to direct measurements of the relevant species of AMP, there are data that are inconsistent with this view. For example, CK-deficient muscle exhibits higher rates of glycogenolysis during intense repeated contractions despite markedly lower levels of P_i_ and phosphorylase **a** (van Deursen et al. 1994; Katz et al. 2003). Moreover, kinetic studies of phosphorylase **b** from CK-deficient muscle demonstrated a higher affinity for AMP compared with muscle from wild-type mice. All these findings were consistent with a key role for AMP in explaining the higher rate of glycogenolysis in CK-deficient muscle. However, accumulation of IMP was significantly lower in CK-deficient vs. wild-type muscle (Katz et al. 2003), which did not support the hypothesis described above. One factor that may have contributed to lower rates of IMP accumulation was the markedly lower activity of AMP deaminase in CK-deficient muscle (Katz et al. 2003; Tullson et al. 1998). Another finding that is relevant in the present context is the study of AMP deaminase-deficient patients. One would expect higher AMP transients in such patients during intense exercise, considering that maximal activities of CK and adenylate kinase are normal (Fishbein et al. 1978, 1985), and assuming that the kinetic properties of the latter two enzymes are unaltered. AMP deaminase-deficient patients and healthy controls performed an ischemic isometric contraction to fatigue at 50% of maximal voluntary contraction force. Contraction duration was similar in the two groups. Biopsies were taken before and after exercise. ATP decreased during contraction and the decrease corresponded to a stoichiometric increase in IMP (about 3 mmol/kg dry wt – recalculated from presented data) in healthy controls, whereas no changes were observed in ATP or IMP in the patients. In contrast, lactate levels (similar in basal state) increased to high and comparable values at fatigue in both groups (Sinkeler et al. 1987). These findings indicate similar rates of glycogenolysis and are not consistent with the idea that AMP transients are responsible for activation of phosphorylase and glycogenolysis during intense exercise.

### In vitro vs. in vitro

Another factor that requires consideration in understanding the control of phosphorylase and glycogenolysis are the values for phosphorylase activity and kinetic constants obtained on purified preparations or dilute extracts in vitro and applying these results to living muscle. First, phosphorylase can be measured in vitro in both directions. However, the activity in the direction of glycogen breakdown is typically only one-third of the activity observed in the direction of glycogen synthesis (Cori et al. 1943; Hanes and Maskell 1942; Danforth and Lyon 1964). In the cell, it is believed that phosphorylase functions only in the direction of glycogen breakdown (Harris et al. 1976). Therefore, this activity will be considered in the following discussion. Assays of human skeletal muscle using saturating levels of substrates (50 mM P_i_), or calculating to saturating values of P_i_ at 35° and at pH 6.8–7.0, yield values of about 150 mmol/min/kg dry wt (Harris et al. 1976; Chasiotis 1983). The temperature and pH reflect those in the muscle at onset of exercise (Harris et al. 1976). Such activities are in excellent agreement with glycogenolytic rates obtained during maximal voluntary isometric contraction in humans (~ 150 mmol/min/kg dry wt) (Ahlborg et al. 1972), as pointed out earlier (Harris et al. 1976). However, one should consider that during 3 s of contraction (maximum duration that 100% force can be maintained), PCr will decrease by about 50% (Ahlborg et al. 1972). If one assumes a resting value of PCr to be 76 mmol/kg dry wt (Harris et al. 1974), then the decrease will be 38 and correspond to an equal increase in the concentration of P_i_ (the trapping of P_i_ in sugar phosphates will be ignored at present). Converting 38 mmol/kg dry wt to mM yields a value of 11.5 mM. Assay of phosphorylase (**a** + **b**) at 11 mM P_i_ yields an activity that amounts to ~ 90 mmol/min/kg dry wt (Chasiotis 1983). And when accounting for loss of P_i_ in accumulation of sugar phosphates, as well as a loss of substrate owing to acidification that occurs as contraction progresses beyond 3 s at lower exercise intensities (HPO_4_^2−^ + H^+^  ↔ H_2_PO_4_^−^, where HPO_4_^2−^ is the substrate) (Chasiotis 1983), the activity will be even lower. It could be argued that the increase in P_i_ during contraction should be added to the concentration present at rest. There is a debate on what the true resting P_i_ concentration is, but suffice it to say that it will range from 1 to 12 mM (Chasiotis 1983). However, since the glycogenolytic rate in skeletal muscle at rest is negligible, then it can be assumed that the concentration of P_i_ will not contribute to the activation of phosphorylase. Later studies demonstrated glycogenolytic rates of ~ 150 and 250 mmol/min/kg dry wt in type I and II human muscle fibers, respectively, during maximal treadmill sprinting over 30 s (Greenhaff et al. 1994). In contrast, the maximal activities of total phosphorylase in human type I and II fibers are, respectively, ~ 70 and 175 mmol/min/kg dry wt under optimal conditions (Harris et al. 1976). When subjects performed maximal sprint cycling over 6–10 s, glycogenolytic rates of 260–430 mmol/min/kg dry wt were recorded in mixed muscle (Gaitanos et al. 1993), which, again, is markedly higher than the measured V_max_ for total phosphorylase in human muscle. Recently, it was demonstrated that stimulation of isolated mouse extensor digitorum longus (type II) muscle at 120 Hz for 1 s at 30 °C yielded a rate of glycogenolysis (based solely on accumulation of glucose 6-P and lactate) of ~ 1150 mmol/min/kg dry wt (Blackwood and Katz 2019)! In the latter study, phosphorylase activity was measured in the direction of glycogen synthesis under optimal conditions at 30 °C yielding an activity of ~ 300 mmol/min/kg dry wt, which would correspond to an activity of only 100 in the direction of glycogen breakdown. These experiments demonstrate a large disconnect between the maximal rates of phosphorylase measured in dilute extracts vs. the high rates of glycogenolysis achieved in living muscle under extreme conditions.

It follows from the discussion above that questions may also arise as to the applicability of kinetic constants derived from in vitro conditions to those in a living cell, as recognized earlier by Cori (1956). Sols and colleagues have amply stressed and addressed this issue in a series of investigations (Aragon et al. 1980a; Aragon and Sols 1991; Lazo and Sols 1980). A clear example of this issue is the observation that phosphofructokinase, a key enzyme of glycolysis, exhibits considerably more activity in situ than in vitro at physiologic concentrations of effector metabolites (Aragon et al. 1980a; Aragon and Sols 1991). Thus, other approaches are required to assess the regulation of phosphorylase either in its natural environment or a simulation of its natural environment. With respect to the latter, use of polyethylene glycol to increase protein (enzyme) concentration, or cross-linking proteins with bifunctional reagents, which will prevent loss of enzymes from the cell, followed by cell permeabilization, have been used to study regulation of enzymes under conditions more closely resembling those in the living cell (Aragon et al. 1980a; Aragon and Sols 1991; Lazo and Sols 1980). An alternative approach is to permeabilize cells with detergents such as saponin, without significantly altering the structure of the protein/organelle of interest. This technique results in the formation of small “holes” in the cell membrane by reacting with cholesterol moieties and is usually used to study mitochondrial function (Kuznetsov et al. 2008). Permeabilization of the cell membrane results in the loss of soluble components from the cytosol (including ligands and proteins) and allows exposure of the mitochondria to pre-determined concentrations of substrates and activators in the medium (Kuznetsov et al. 2008). In an earlier study, saponin was applied to isolated hepatocytes for 10 min, and there was no loss of phosphorylase activity (Burgess et al. 1983), probably because phosphorylase is associated with glycogen in a glycoprotein complex (Heilmeyer et al. 1970) that is too large to pass through the permeabilized cell.

**Fig. 1:**
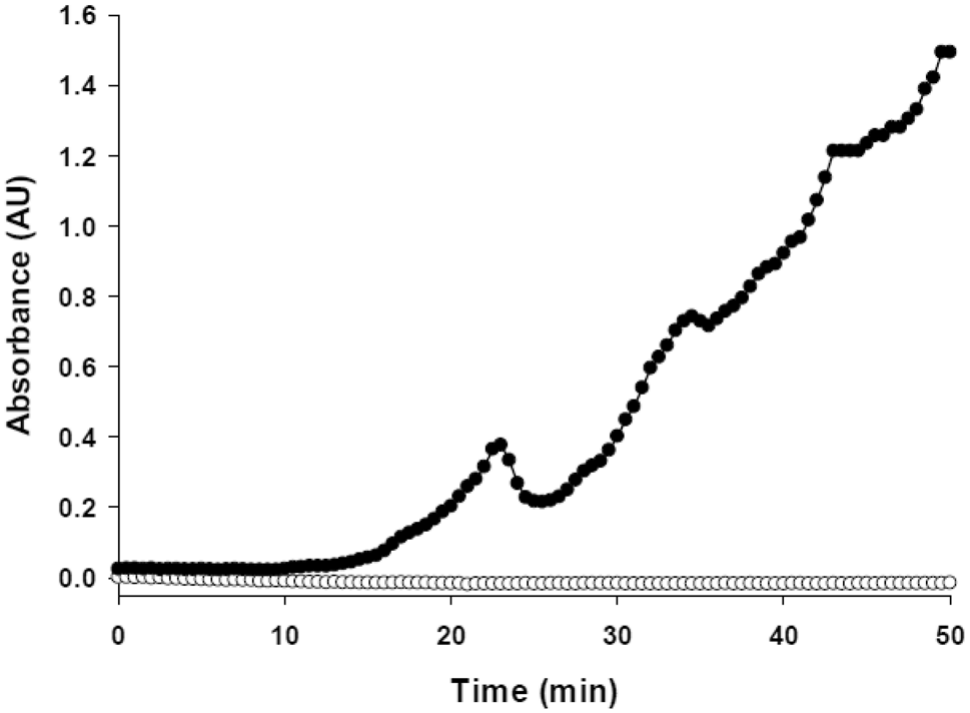
Phosphorylase activity in a permeabilized human muscle bundle (0.43 mg dry muscle). Absorbance is measured at 340 nm at 25 °C (filled circle, bundle; unfilled circle, blank). Medium contained P_i_ (20 mM), glycogen (70 mM as glucosyl units) and AMP (2 mM), pH 7.0. Note the linear activities with a periodicity of ~ 10 min. Peak phosphorylase activity amounted to 47 µmol/min/g dry muscle (unpublished observations by Blackwood and Katz)

We, therefore, attempted to measure phosphorylase activity in a saponin-permeabilized human muscle (m. vastus lateralis) bundle. A biopsy was taken from a subject after an overnight fast (glycogen values assumed to be high). The biopsy was divided into several aliquots. One aliquot was immediately frozen in liquid nitrogen, freeze-dried and processed for analysis of total phosphorylase activity in the direction of glycogen breakdown (control value = 75 mmol/min/kg dry wt at 25 °C). Three aliquots were exposed to saponin for 30 min, frozen, freeze-dried and assayed for phosphorylase (to assess how much phosphorylase was lost during permeabilization). The amount of phosphorylase remaining in the fiber after exposure to saponin for 30 min ranged from 72 to 80% (mean = 77%). These results demonstrate that most of the phosphorylase remains within the muscle after permeabilization. A biopsy from another subject was permeabilized and then placed in 1 ml of phosphorylase assay mixture (20 mM P_i_, 70 mM glycogen as glucosyl units, 2 mM AMP, pH 7.0 with phosphoglucomutase and glucose 6-P dehydrogenase and NADP^+^ at 25 °C) and placed in a spectrophotometer for 50 min. After a lag of about 10 min (possibly the time required for substrates and AMP to orient correctly with phosphorylase in fibers), activity was detected (Fig. 1). There appeared to be a burst of activity approximately every 10 min. The reason for this is not clear but it is reminiscent of the glycolytic oscillations observed earlier in skeletal muscle cell-free extracts (Tornheim et al. 1991). The activity of total phosphorylase amounted to 47 mmol/min/kg dry wt at 25 °C. What is particularly noteworthy is that the (soluble) protein concentration was 500 µg/ml incubation medium, whereas we normally use about 5–10 µg/ml for measuring activity in dilute extracts in the linear range. The system has not been optimized yet but it raises the possibility of a new approach to study the regulation of phosphorylase under more physiologic conditions.

### Other modes of regulation

Other modes of covalent regulation of phosphorylase have recently been described. Acetylation of liver phosphorylase results in inhibition of phosphorylase and promotes dephosphorylation of phosphorylase **a** as well (Zhang et al. 2012). However, to the author’s knowledge, such a mechanism has not been demonstrated in skeletal muscle. Oxidation and nitration of phosphorylase purified from skeletal muscle have been demonstrated, albeit nitration is much more inhibitory than oxidation (Mathieu et al. 2016; Dairou et al. 2007). In contrast, oxidation of brain phosphorylase is very potent, but without effect on liver phosphorylase (Mathieu et al. 2016). Oxidative stress and nitration have also been shown to inhibit phosphorylase activity in cell cultures (Mathieu et al. 2016; Dairou et al. 2007). Recent studies also demonstrated mild inhibition by oxidation (H_2_O_2_) and potent inhibition by nitration (peroxynitrite) of phosphorylase activity in dilute mouse muscle extracts, which were fully reversed by dithiothreitol (Blackwood et al. 2021). Moreover, incubation of isolated intact mouse muscle preparations with high concentrations of peroxynitrite resulted in nitration of phosphorylase and marked inhibition of glycogenolysis during short-term intense, repeated contractions. In contrast, repeated contractions in the absence of exogenous peroxynitrite did not result in nitration of phosphorylase, nor did the addition of exogenous antioxidants alter glycogenolysis during repeated contractions. These data suggest that in the presence of excessive levels of nitrating/oxidizing agents, phosphorylase and glycogenolysis may be inhibited in skeletal muscle, but during intense contractions in living muscle under physiological conditions nitration/oxidation probably does not regulate phosphorylase activity and glycogen breakdown.

### Contraction

What is apparent from the discussion above is that some factor associated with the contraction process is primarily responsible for the activation of phosphorylase and high rates of glycogenlysis. Early on, Cori demonstrated that there is a correlation between the rate and amount of work performed and the degree to which phosphorylase and glycogenolysis are activated during contraction (Cori 1956). At that time, no attempt was made to interfere with tension development during the contraction process to further study the relationship between force generation and glycogenolysis. Subsequent studies, however, raised questions regarding the correlation reported above. For example, prior stretching of isolated rat soleus (type I, slow twitch) muscle preparations to a length that precluded actomyosin interactions, thereby blocking active force generation during repeated contractions induced by electrical stimulation, had little effect on glycogenolysis vs. maximal tension development conditions (Ihlemann et al. 1999). In an alternative approach, N-benzyl-p-toluene sulphonamide (BTS), an inhibitor of myosin II ATPase, was used to block force generation during repeated contractions of isolated mouse extensor digitorum longus (type II, fast-twitch) induced by electrical stimulation. Whereas BTS blocked ~ 95% of tension development during repeated contractions, glycogenolysis was diminished only by ~ 25% vs. contractions in the absence of BTS (Sandstrom et al. 2007; Zhang et al. 2006). The latter findings indicate that tension development/cross-bridge cycling is not required to reach high rates of glycogenolysis during intense short-term contractions in isolated muscle preparations.

**Fig. 2:**
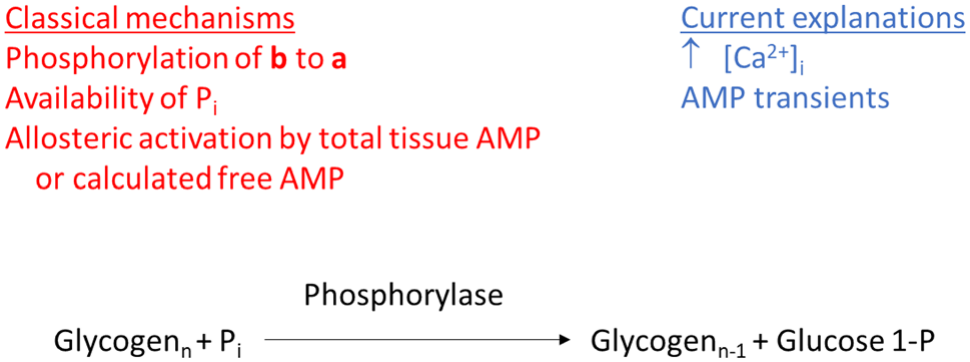
Mechanisms of phosphorylase-mediated breakdown of glycogen. Vertical arrow denotes increase. As discussed in text, none of the classical mechanisms or current explanations have been demonstrated experimentally to account for the high rates of glycogenolysis in living muscle

From the above discussion, we may summarize the following. Simply elevating levels of phosphorylase **a** and P_i_ is not sufficient to achieve high rates of glycogenolysis in skeletal muscle. At the onset of intense short-term contractions, conversion of phosphorylase **b** to **a** may play an important role in contributing to activation of glycogenolysis (assuming the presence of an activating factor). Unfortunately, use of phosphorylase **b** kinase-deficient muscle does not result in conclusive findings on this point. This issue may be addressed in the future by use of either specific inhibitors of phosphorylase **a** or genetic manipulation resulting in a phosphorylase isoform that is not recognized by phosphorylase **b** kinase. For most forms of exercise/contractions that exceed a duration of several seconds, it appears that activation of phosphorylase **b** is primarily responsible for glycogenolysis. The activation of phosphorylase **b** is associated with a high rate of energy turnover during the contraction process, but does not appear to require substantial degrees of force generation/cross-bridge cycling. While increases in myoplasmic Ca^2+^ and/or relevant species of AMP may account for the activation of phosphorylase, there is no conclusive evidence that this is the case (Fig. 2). One approach to test the importance of AMP would be to generate an isoform of phosphorylase that is insensitive to AMP, as suggested earlier (Katz and Westerblad 2014). Thus, the mechanism to explain high rates of glycogen breakdown in living muscle remains to be established.

### Glycogen synthase and glycogenolysis during exercise

Net glycogen degradation is a function of two opposing enzyme activities: phosphorylase and GS. Since maximal in vitro total phosphorylase activity is usually about 20–30-fold higher than total in vitro GS activity (depending on whether phosphorylase is measured in the forward or reverse direction) (Chasiotis 1983; Katz 1997), it appears that the contribution of GS to control of net decreases in glycogen will probably be minor, especially during conditions of high-intensity exercise. During certain conditions, such as low intensity exercise combined with administration of glucose (which will raise both circulating glucose and insulin values), glycogen levels can increase, but it appears that the increase will be primarily confined to less active type II muscle fibers (Kuipers et al. 1987; Hultman et al. 1971). Still regulation of GS during exercise is of interest in terms of understanding the simultaneous regulation of the first two enzymes reported to be controlled by reversible covalent phosphorylation, albeit in opposite manners (i.e., phosphorylation results in activation of phosphorylase and inhibition of GS). The effects of exercise/muscle contraction on changes in GS activity and its regulation in terms of phosphorylation, substrate availability, allosteric regulation and changes in cellular localization were described and reviewed earlier (Nielsen and Richter 2003). Suffice it to say at present that during intense short-term exercise, or during the initial stages of prolonged exercise, GS is inactivated (phosphorylated). As exercise progresses and glycogen levels decrease, the enzyme becomes dephosphorylated (activated) and the degree of activation (dephosphorylation) can exceed basal levels by the end of exercise. Within minutes after exercise, a further dephosphorylation occurs. The latter is likely due to decreases in the activities of protein kinases that were activated during exercise (e.g., cAMP dependent protein kinase) (Yan et al. 1992). Moreover, at the onset of exercise, the allosteric activator (glucose 6-P) will increase substantially and then decrease by the end of prolonged exercise (Katz et al. 1991b). Finally, although GS will be highly dephosphorylated by the end of prolonged exercise, the enzyme will be destabilized owing to glycogen depletion (glycogen is required to stabilize and maintain GS activity) (Jiao et al. 1999). Thus GS is not likely to play a significant role in control of net glycogen degradation during exercise. However, the activation (dephosphorylation) of GS during the latter phase of prolonged moderate exercise may be viewed as a preparation for optimizing glycogen storage immediately after termination of exercise (see below).

## Regulation of glycogen synthesis in living muscle during recovery from contraction/exercise

### Glucose, insulin and supercompensation

During recovery from muscle contractions/exercise glycogen accumulation occurs at rates that depend on the experimental conditions. For example, in isolated muscle preparations, glycogen accumulation depends on the availability of glucose in the medium (Chin and Allen 1997; Helander et al. 2002). Moreover, if insulin is added in the presence of glucose, then the rate of glycogen synthesis and glycogen accumulation are enhanced (Richter et al. 1982; Ivy and Holloszy 1981). Similarly, following exercise in humans, ingestion of carbohydrate enhances glycogen synthesis, and additional elevations of plasma insulin result in further increases of muscle glycogen levels (Bergstrom et al. 1967; Zawadzki et al. 1992; Ivy et al. 1988). Accumulation of glycogen in humans appears to follow two phases: 1. an initial, rapid phase that is independent of insulin and enhanced by low glycogen levels; and 2. a subsequent, slower more prolonged phase that is dependent on insulin (Price et al. 1994). The initial rate of muscle glycogen accumulation following ingestion of carbohydrate in humans during the first 2 h of recovery is on the order of 0.5 mmol glucosyl units/min/kg dry wt (Ivy et al. 1988; Maehlum et al. 1977). The activity of human muscle GS (adjusted to 35 °C assuming a Q_10_ of 2) is ~ 11 mmol/min/kg dry wt at saturating glucose 6-P, and by the end of exercise the activity ratio is about 50% (activity measured at 0.17/7.2 mM glucose 6-P), and at that time the concentrations of glucose 6-P and UDP-glucose are ~ 250 µM (Katz et al. 1991b; Yan et al. 1993). If the kinetic constants obtained in vitro (see above) are applicable to living muscle, then there is sufficient enzyme activity to account for the formation of glycogen. If adequate ingestion of carbohydrate is continued for several days following exercise that results in sufficient degradation of glycogen, then muscle glycogen levels will exceed baseline values, i.e., glycogen supercompensation (Bergstrom et al. 1967; Bergstrom and Hultman 1966b; Kochan et al. 1979). The phenomenon of supercompensation is localized to the muscle that underwent prior glycogen depletion (Kochan et al. 1979; Bergstrom and Hultman 1966b). Traditionally, it has been ascribed to sustained activation of GS rather than diminished glycogen breakdown (Hultman et al. 1971; Kochan et al. 1979). Recent findings indicate that, in addition to activation of GS, activation of an isoform of AMP-dependent protein kinase (AMPK, α1β2γ1) is also required to achieve supercompensation in skeletal muscle (Hingst et al. 2018). It was suggested that the increased activity of the α1 isoform of AMPK enhanced fatty acid oxidation and inhibited carbohydrate oxidation, thereby, channeling more glucose toward glycogen synthesis. However, no evidence was presented that muscle fat oxidation would be elevated and carbohydrate oxidation diminished 2–3 days after glycogen depleting exercise while on a high carbohydrate diet.

### Glycogenin and glycogen storage

With the discovery of glycogenin, a renewed interest was kindled in understanding control of glycogen biogenesis, as well as its role in supercompensation after exercise, since it was initially considered to set the upper limit for glycogen storage in muscle (Kennedy et al. 1985; Smythe and Cohen 1991). However, even after depletion of glycogen to low/barely detectable levels following prolonged submaximal exercise, glycogenin remained sufficiently glucosylated to preclude measurement of enzyme activity, as measured by autoglucosylation or protein expression unless preceded by amylolysis (Jiao et al. 1999). This observation seemed to preclude an important role for glycogenin in regulating glycogen synthesis after acute exercise. Subsequently, others reported a lack of relationship between glycogenin expression and glycogen content as assessed in different muscle fiber types or between trained and untrained muscle in rats (Hansen et al. 2000). Moreover, overexpression of glycogenin did not result in increases in glycogen content in fibroblasts (Skurat et al. 1997, 1996). In experiments using primary rat myotubes in culture, glycogen was depleted by electrical stimulation or hypoxia (Mamedova et al. 2003). Thereafter, cells recovered for up to 120 h in the presence of glucose. Marked glycogen supercompensation was observed in both experimental models by 120 h vs. control (2.5- and fourfold increases after electrical stimulation and hypoxia, respectively). Interestingly, glycogenin protein levels were increased during supercompensation in both treatments (~ twofold). However, increases in other factors that could contribute to glycogen supercompensation were also observed (e.g., increased expression of GLUT4 protein and activation of GS) (Mamedova et al. 2003), and no interventions were attempted to isolate the role of glycogenin. Overexpression of constitutively active GS in mice also results in marked increases in muscle glycogen content that is also associated with increases in glycogenin expression, but branching is decreased (Pederson et al. 2003).

Recently, a new glycogen storage disease was described wherein a mutation in glycogenin resulted in an inactive protein that was associated with lack of glycogen storage in skeletal muscle (GSD XV) (Moslemi et al. 2010). Subsequent studies described patients with mutations in glycogenin or glycogenin deficiency who store glycogen, as well as polyglucosan in muscle (Hedberg-Oldfors et al. 2019; Krag et al. 2017; Visuttijai et al. 2020). The glycogen is degradable as evidenced by an increase in blood lactate during exercise, albeit the increase is less than that observed in control subjects (Stemmerik et al. 2017). Humans express two isoforms of glycogenin: 1 and 2, that are coded for, respectively, by GYG1 and GYG2 (Roach et al. 2012). To account for the presence of glycogen in the absence of glycogenin-1, it was postulated that glycogenin-2 could function as an alternative primer for glycogen biogenesis (Moslemi et al. 2010). Indeed, an increased expression of glycogenin-2 was described in patients who lacked glycogenin-1 in skeletal muscle (Krag et al. 2017). A subsequent study, however, could not demonstrate an increased expression of glycogenin-2 in skeletal muscle that lacked glycogenin-1 (Visuttijai et al. 2020). The latter investigators made the important observation that in the earlier study where glycogenin-2 was found to be upregulated, no evidence was presented that the protein was functional. It was concluded that glycogenin was dispensable for glycogen synthesis in human muscle (Visuttijai et al. 2020). In a further attempt to examine the role of glycogenin in skeletal muscle biogenesis, glycogenin knockout mice were generated (Testoni et al. 2017). Contrary to expectation, not only was glycogen biogenesis not blocked, but glycogen levels markedly exceeded those in wild-type muscle. Moreover, the glycogen was normally branched, fully degraded by amylase and was broken down during exercise, indicating that the glycogen was of normal structure. Finally, the glycogen was free of covalently bound protein, demonstrating that a protein primer was not required for glycogen biogenesis. The authors hypothesized that glycogen biogenesis in the glycogenin knockout mice may begin with free glucose, as described above, resulting in the formation of maltose, which could also serve as a substrate for GS (Testoni et al. 2017). However, the extremely high K_m_ values for these reactions, as determined under in vitro conditions (Salsas and Larner 1975; Goldemberg 1962), raise questions regarding their likelihood in vivo. The investigators also provided the novel suggestion that the function of glycogenin may be to limit glycogen storage. A strict test of this hypothesis would entail controlling for many other factors involved in control of glycogen levels. Noteworthy is that the investigators also generated a mouse model expressing a skeletal muscle form of GS that was resistant to inactivation by phosphorylation. The muscle glycogen levels achieved in these mice were even higher than those obtained in the glycogenin deficient mice. However, the expression of glycogenin was also markedly higher in the muscle of mice expressing the modified form of GS (Testoni et al. 2017). This observation is inconsistent with the idea that glycogenin limits glycogen storage. Alternatively, the increase in glycogenin expression is a compensatory response to ensure that glycogen does not increase even further. In conclusion, the true function of glycogenin in skeletal muscle remains unclear.

### Phosphorylase and glycogen storage

Although it was originally believed that phosphorylase was responsible for glycogen synthesis, demonstration of the high P_i_/glucose 1-P ratio (75- to 100-fold removed from phosphorylase equilibrium), together with discovery of GS and UDP-glucose pyrophosphorylase in skeletal muscle, indicated that glycogen synthesis in the cell could only occur via the latter two enzymes (Larner et al. 1960; Villar-Palasi and Larner 1958). The final evidence was provided by the observation of high glycogen content in muscle in the absence of phosphorylase activity (McArdles disease, GSD V), but essentially normal activities of UDP-glucose pyrophosphorylase and GS (Larner and Villar-Palasi 1959; Schmid et al. 1959). While these findings appear to preclude a role for phosphorylase in active glycogen synthesis, they do not rule out a role for phosphorylase in glycogen storage, as has been described in liver (Massillon et al. 1995; Aiston et al. 2001).

**Fig. 3:**
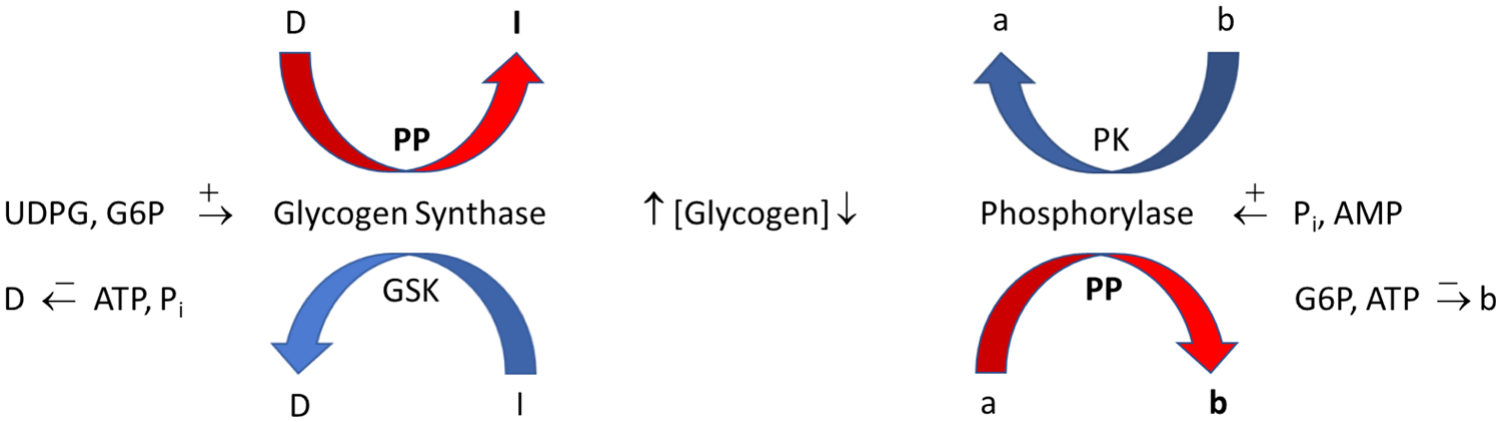
Regulation of glycogen accumulation during recovery from exercise. UDPG, UDP-glucose; D, phosphorylated form of glycogen synthase (GS) that is dependent on glucose 6-P (G6P); I, non-phosphorylated GS that is independent of G6P; a, phosphorylated phosphorylase that is independent of AMP; b, non-phosphorylated phosphorylase that is dependent on AMP; PP, protein phosphatases; PK, phosphorylase kinase; GSK, GS kinases; arrow with positive sign ( +) indicates activation of enzyme; arrow with negative sign ( − ) indicates inhibition of D or b form of enzyme. Bold **PP**, **I**, and **b** and red arrows indicate that this is the process that predominates during recovery from exercise. The bold vertical upward arrow indicates that glycogen concentration is increasing under these conditions. See text for further details

Glycogen cycling refers to the simultaneous occurrence of glycogen synthesis and breakdown. The observation that glycogen cycling occurs in isolated muscle at rest demonstrates that phosphorylase is active, but is balanced by the activity of GS (Challiss et al. 1987). We now return to the observation that phosphorylase is inactivated (dephosphorylated, after initial phosphorylation) after intense short-term, as well as after prolonged exercise in humans; similar findings are observed in rodent muscle after intense short-term swimming and in isolated muscle following contractions induced by electrical stimulation (Chasiotis et al. 1982; Piras and Staneloni 1969; Conlee et al. 1979; Jiao et al. 1999; Brau et al. 1997). Dephosphorylation continues during the initial recovery period in human muscle and in isolated mouse muscle (Jiao et al. 1999; Sandstrom et al. 2004), and in rat skeletal muscle, myotubes and heart cells in culture dephosphorylation can continue for up to 5 days following glycogen depletion (Mamedova et al. 2003; Vigoda et al. 2003). In intact rats, activity of phosphorylase **a** increases continuously back to baseline over a 2 h recovery period (Brau et al. 1997). The dephosphorylated enzyme undergoes limited phosphorylation in response to subsequent electrical stimulation or exposure to epinephrine and this phenomenon appears to have little relation to the glycogen content (Constable et al. 1986). Thus, intense contractions result in a marked and persistent dephosphorylation of phosphorylase during recovery from exercise. The significance of this phenomenon is not fully understood, but it has been suggested to play an important role in the replenishment of glycogen following exercise/glycogen depletion (Sandstrom et al. 2004; Mamedova et al. 2003; Vigoda et al. 2003; Brau et al. 1997). In recent studies, the quantitative contribution of phosphorylase, owing to its inactivation/activation during recovery from repeated contractions, to glycogen accumulation was calculated to range from 45 to 75% in isolated mouse fast- (extensor digitorum longus) and slow-twitch (soleus) muscle (Blackwood et al. 2018; Blackwood et al. 2019). An earlier study of isolated fast-twitch muscle (epitrochlearis) following prolonged treadmill running in rats demonstrated negligible increases in the rate of glycogen synthesis but more than 50% inhibition of glycogenolysis compared with the basal condition (Challiss et al. 1987), which reflects the importance of exercise in the quantitative inhibition of phosphorylase. Under these conditions, one would expect to see accumulation of glycogen during recovery (Constable et al. 1986). Noteworthy is that the control of phosphorylase during recovery from exercise need not be limited to control by phosphorylation but can also be attributed to changes in availability of P_i_. This is illustrated by the observation that during recovery from repeated contractions in isolated mouse muscle preparations, increasing temperature of the incubation medium from 25 to 35 °C retards glycogen accumulation and this is associated with elevated levels of P_i_ (Blackwood et al. 2018; Blackwood et al. 2019). One should consider that the rate of glycogen accumulation in isolated skeletal muscle during recovery from repeated contractions in the absence of insulin is generally < 0.5 mmol glucosyl units/min/kg dry wt (Sandstrom et al. 2004; Blackwood et al. 2018; Blackwood et al. 2019). At these low rates, small changes in phosphorylase activity can have marked effects on glycogen storage, considering that phosphorylase activity is generally > tenfold greater than GS activity in skeletal muscle (see below). A scheme of how activation of GS and inhibition of phosphorylase during recovery from exercise is presented (Fig. 3).

The above discussion leads to the question of whether phosphorylase inactivation is also important under other conditions associated with glycogen storage in skeletal muscle. Insulin stimulates GS (dephosphorylates), glycogen synthesis as well as glycogen accumulation, but, in general, does not alter phosphorylase fractional activity in isolated muscle or in vivo (LeMarchand-Brustel and Freychet 1979; Le Marchand-Brustel and Freychet 1981; Craig and Larner 1964; Torres et al. 1968). It should be noted that phosphorylase fractional activity in carefully isolated muscle preparations is generally low, and therefore, dephosphorylation by insulin may be difficult to detect. However, in an earlier study, cellular phosphoproteins in isolated rat muscle (epitrochlearis) preparations were pre-labeled with ^32^P_i_ before treatment with insulin (Zhang et al. 1989). Thereafter, phosphorylase was immunoprecipitated and the amount of phosphate incorporated into the enzyme was measured and found to be diminished by 50% following exposure to insulin. Remarkably, the investigators also succeeded in measuring a significant decrease in phosphorylase fractional activity in response to insulin (from 15 to 11%), albeit the decrease was smaller than the decrease observed in phosphorylation. Significantly, measurements of glycogen cycling in isolated muscle demonstrate that at a saturating concentration of insulin (1 mU/L), glycogen synthesis increases ~ threefold while inhibition of glycogenolysis is ~ sevenfold (Challiss et al. 1987). Taken together, the results data indicate that phosphorylase inactivation is also of quantitative significance for glycogen formation in response to insulin, as recognized earlier (Zhang et al. 1989).

## Role of glycogen in skeletal muscle

Although glycogen is well recognized as an energy substrate, it has also been suggested to serve as a P_i_ trap, play a role in sarcoplasmic reticulum (SR) dependent Ca^2+^ release and muscle excitability, as well as regulate K^+^ homeostasis, enzyme activity, gene expression, translational and posttranslational processes (Drahota et al. 1958; Philp et al. 2012; Vigh-Larsen et al. 2021; Blackwood and Katz 2019). Within the scope of this review, only the acute role of glycogen in muscle energetics and force generation will be addressed.

### Glycogen and fatigue

Glycogen is likely the major energy substrate during most forms of physical exercise, especially during moderate (≥ 50% of maximal oxygen uptake ((VO_2_max)) to heavy activities lasting > 20 s (Hultman and Sjoholm 1983a). It has long been recognized that glycogen availability limits exercise endurance in humans at intensities corresponding to 60–80% of VO_2_max (Bergstrom et al. 1967; Hermansen et al. 1967), which generally includes activities such as running, cycling, skiing, etc. This observation has led to strategies to increase muscle glycogen levels prior to performance testing/competitions (i.e., glycogen supercompensation/loading). Initially, glycogen supercompensation was achieved by first depleting muscle stores by heavy exercise and then resting for several days while ingesting a diet rich in carbohydrate (Bergstrom and Hultman 1966b). Subsequent studies showed that optimal supercompensation could be achieved by performing two exhaustive bouts of exercise separated by 3 days of a low carbohydrate diet, followed by 3 days of a high carbohydrate diet and rest (Hultman 1967). Later studies showed that supercompensation could also be achieved with a more moderate exercise-diet regimen (Sherman et al. 1981).

It has also been established that glycogen limits force production during repeated contractions in isolated muscle preparations (Chin and Allen 1997; Helander et al. 2002; Kabbara et al. 2000). Probably one of the most convincing arguments in support of the importance of glycogen for muscle performance is the premature fatigue associated with McArdles disease (GSD V), where there is a lack of glycogen phosphorylase (Lewis and Haller 1986) or with glycogen synthase deficiency (GSD 0b), where there is a lack of glycogen (Kollberg et al. 2007; Sukigara et al. 2012). However, while the link between glycogen and fatigue has been established under defined conditions, the cellular events that lead to the loss of force generation are still not fully understood. Several explanations have been suggested, including substrate limitation for production of acetyl-CoA or anaplerosis (expansion of the tricarboxylic acid cycle pool) (Sahlin et al. 1990b). In the absence of adequate carbohydrate stores, net ATP degradation occurs as reflected by increases in IMP, demonstrating energetic stress (Sahlin et al. 1990b; Norman et al. 1987; Broberg and Sahlin 1989). An additional explanation is that glycogen is required to maintain adequate SR-mediated Ca^2+^ release to activate cross-bridges. Glycogen has been suggested to affect this process by separate mechanisms. In one scenario, glycogen localized to the SR/T-tubular complex has been suggested to bind to the ryanodine receptor or bridge the dihydropyridine/ryanodine receptor region (Kabbara et al. 2000; Stephenson et al. 1999; Barnes et al. 2001; Nielsen et al. 2009). In this case, glycogen would play a structural role independent of its function as an energy source. It should be noted, however, that direct evidence in support of such a mechanism has not been presented. Alternatively, the localized glycogen pool has the enzymatic capacity to generate ATP, thereby activating ATP-sensitive sites on the ryanodine receptor, as well as minimizing accumulation of ATP degradation products (ADP and AMP), which will also increase the open probability of the SR Ca^2+^ release channels (Entman et al. 1980; Han et al. 1992; Laver et al. 2001; Meissner et al. 1986). Finally, under many conditions, inhibition of cross-bridges/force generation is attributed to accumulation of P_i_ (Allen et al. 2008). Glycogen has been suggested to attenuate increases in P_i_ by minimizing metabolic stress and by trapping the metabolite in hexose phosphates (Katz and Westerblad 2014; Blackwood and Katz 2019). More in-depth discussions on how glycogen affects muscle performance are presented elsewhere (Vigh-Larsen et al. 2021; Katz and Westerblad 2014; Ortenblad et al. 2013).

However, the role of glycogen in muscle fatigue is not unchallenged. Knockout of GS in skeletal muscle of mice resulted in lack of detectable glycogen levels, but no effect on liver glycogen content (GS in skeletal muscle is coded for by the GYS1 gene and in liver by the GYS2 gene (Roach et al. 2012)). Only about 10% of such mice survive birth, apparently due to impaired cardiac function (Pederson et al. 2004, 2005b). Surprisingly, treadmill running performance of GS-deficient mice was not negatively affected. The authors suggested that blood-borne glucose, derived from hepatic glycogenolysis, was sufficient to maintain the energetic requirements for muscle performance (Pederson et al. 2005b). Generally, this appears to be the case in rodents, i.e. a greater reliance on liver vs. muscle glycogen (for references see (Pederson et al. 2005b)). An important point to note in the latter study is that muscle glycogen levels in fed wild-type mice was ~ 6 µmol/g wet in hindlimb muscles (Pederson et al. 2005b), which is very low, even though a clear decrease to < 1 µmol/g wet wt was observed after exercise. If liver glycogen is depleted prior to exercise, then a role for muscle glycogen in fatigue becomes more apparent (Pederson et al. 2005b). In a subsequent report, expression of GS in mouse skeletal muscle was decreased to ~ 15% and muscle glycogen decreased to ~ 30% of the values observed in wild-type mice (~ 25 µmol/g wet wt), whereas liver glycogen levels were markedly increased (Xirouchaki et al. 2016). In contrast to the earlier report, muscle performance during treadmill running in the latter study was markedly decreased and lactate accumulation was substantially increased (Xirouchaki et al. 2016). While several potential explanations were discussed, the one that seemed most likely to explain the divergent results was the marked decrease in expression of hexokinase II in muscle of the GS knockout mice (Xirouchaki et al. 2016). Insufficient glucose phosphorylation should result in a metabolic stress that would lead to an activation of phosphorylase (see above). In this scenario, one would expect a lower rate of glucose uptake, and higher rates of glycogenolysis and lactate accumulation. Unfortunately, the changes in muscle glycogen during exercise were not reported, whereas a decreased capacity for glucose uptake and increased lactate accumulation in muscle were indeed confirmed (Xirouchaki et al. 2016). In an alternative approach, it was shown that overexpression of GS in mice resulted in marked increases in skeletal muscle glycogen content. While glycogen degradation was greater in these mice during treadmill running, liver glycogenolysis was decreased and exercise performance was not significantly affected (Pederson et al. 2005a). Taken together, the data suggest that muscle glycogen content does not limit exercise performance in mice. It would be of interest to study muscle performance with appropriate protocols in isolated GS-deficient/GS overexpression muscles to obtain additional information on the importance of glycogen in fatigue.

### Glycogen and force generation

As indicated above, glycogen has been suggested to play an important role in force generation by maintaining SR-mediated Ca^2+^ release (Kabbara et al. 2000; Stephenson et al. 1999; Chin and Allen 1997; Helander et al. 2002; Duhamel et al. 2006). For example, prior depletion of glycogen by repeated contractions, followed by 60–120 min of recovery, results in recovery of low- and high-frequency force generation that is dependent on the availability of glucose in the medium, and, hence, the restoration of glycogen in the muscle (Helander et al. 2002; Chin and Allen 1997; Cheng et al. 2017). Moreover, the recovery of tetanic intracellular Ca^2+^ [Ca^2+^]_i_ exhibits a glycogen-dependent and independent component, where the latter depends on the previous contractile activity (Chin and Allen 1997). Whether glycogen plays a structural role or functions as an energy substrate in recovery of force and [Ca^2+^]_i_ is not clear. However, the putative glycogen-dependent structure has not been demonstrated, and the bulk of available data support a metabolic component (Vigh-Larsen et al. 2021). There is evidence, however, that the restoration of force following recovery after glycogen depletion can be dissociated from glycogen, and this appears to depend on the experimental conditions. In a recent study, repeated contractions of isolated mouse EDL muscles at 25 °C resulted in substantial glycogen depletion (to 15% of basal) and marked inhibition of force generation (< 5% of initial) (Blackwood et al. 2018). Following 120 min of recovery in 5.5 mM glucose, high-energy phosphates and glycogenolytic intermediates (including lactate) were restored to baseline levels and glycogen recovered to ~ 80% of basal. Under these conditions, tetanic isometric force was substantially restored (40–70% of initial force at stimulation frequencies of 30–150 Hz). If, however, temperature was increased to 35 °C during recovery, low frequency force (30 Hz, measured at 25 °C) was markedly increased (~ 35% higher vs. recovery at 25 °C), whereas force at the higher frequencies was not affected. Similar observations were seen in isolated single flexor digitorum brevis muscle fibers and the increased force was associated with a marked increase in tetanic [Ca^2+^]_i_ (Cheng et al. 2017). If this was a glycogen-dependent phenomenon, one would have predicted that glycogen would have been even higher after recovery at 35 °C. Surprisingly, not only was glycogen not elevated, but it was substantially depressed (~ 20% of basal) (Blackwood et al. 2018). This clear dissociation between restitution of force and tetanic [Ca^2+^]_i_ on one hand, and glycogen on the other hand, demonstrates the importance of experimental conditions in describing a phenomenon. In summary, it appears that muscle glycogen is an important energy substrate during prolonged submaximal exercise in humans, whereas liver glycogen appears to be a more important energy substrate in rodents.

### Glycogen and P_i_

As discussed above, P_i_ is considered an important factor in inhibiting cross-bridge force generation, but it also decreases the sensitivity of cross-bridges to Ca^2+^. P_i_ is also implicated in SR-mediated Ca^2+^ release by, on the one hand, enhancing ryanodine receptor-dependent Ca^2+^ release and, on the other hand, inhibiting Ca^2+^ release owing to precipitation of Ca^2+^- P_i_ after entering the SR, especially during the late stages of fatigue. Additional details on P_i_ and muscle force generation are provided elsewhere (Allen et al. 2008). Glycogen has been suggested to maintain force development by attenuating accumulation of P_i_ (Helander et al. 2002; Katz and Westerblad 2014) and this is achieved via its role as an energy substrate. However, glycogen can also decrease P_i_ values by functioning as a P_i_ trap. This would become most apparent during the fight or flight response when there is a massive secretion of adrenaline. Thus exposure of isolated muscle preparations to isoproterenol (β-receptor agonist) or infusion of humans and rats with adrenaline or inhalation by humans of terbutaline (β-receptor agonist) results in a marked conversion of phosphorylase **b** to **a** that is associated with a trapping of P_i_ in accumulated hexose phosphates, resulting in a decrease in free P_i_ (Blackwood and Katz 2019; Kalsen et al. 2016; Chasiotis and Hultman 1985; Chasiotis 1985). This can be viewed as a preparatory phase prior to activation of muscle contraction. In the resting state, the effect of β-receptor agonists on accumulation of hexose phosphates is clear but negligible in comparison with the increases seen during maximal cycling over 10 s in humans. Under these conditions, accumulation of glucose 6-P amounted to ~ 10 mmol/kg dry wt in control and 30 mmol/kg dry wt after inhalation of terbutaline (Kalsen et al. 2016). Unfortunately, no direct measurements of P_i_ were made in the latter study. However, calculations of the expected increases in P_i_ based on accumulation of hexose phosphates and depletion of PCr and ATP indicate that P_i_ increased by 40 mmol/kg dry wt in control and by only 7 after inhalation of terbutaline. Because the capacity to accumulate hexose phosphates during intense short-term exercise is markedly greater in human than in rodent skeletal muscle (Hostrup et al. 2014; Gaitanos et al. 1993; Bogdanis et al. 1998; Dudley and Terjung 1985; Conlee et al. 1979; Crow and Kushmerick 1982; Katz et al. 2003), it is likely that P_i_ trapping would be a more significant mechanism in the former than in the latter. The reason for the difference between humans and rodents is not clear but may relate to glycogen levels (see below).

### Glycogen in human vs. mouse muscle

As mentioned earlier, there are several differences in glycogen metabolism between rodents and humans. Since results from rodent muscle often serve as a basis for understanding glycogen metabolism in humans, a discussion of the differences between species follows. Glycogen may serve different functions in large and small mammals. Indeed, it was recently demonstrated that glycogen metabolism is differentially regulated and, possibly, for different purposes in type I and II muscle fiber types, depending on experimental conditions (Blackwood and Katz 2019). One obvious contrast between human and mouse skeletal muscle, for example, is the marked difference in muscle glycogen content. From perusal of the literature, muscle glycogen levels in the mouse appear to range from 25 to 100 mmol glucosyl units/kg dry wt (Blackwood and Katz 2019; Pederson et al. 2005b; Testoni et al. 2017; Danforth 1964; Xirouchaki et al. 2016), whereas in humans, values generally range from 300 to 600 (Gaitanos et al. 1993; Harris et al. 1974; Hermansen et al. 1967; Yan et al. 1993), and can reach levels of 1000 after glycogen supercompensation (Bergstrom and Hultman 1966b). Thus, human muscle contains ~ 10 times as much glycogen as does mouse muscle, despite similar activities of phosphorylase and GS in the two species as measured under the same assay conditions (Jiao et al. 1999; Katz 1997; Blackwood et al. 2018, 2021; Sandstrom et al. 2004; Katz et al. 1991a). Interestingly, despite marked differences in subsarcolemmal and intermyofibrillar glycogen contents, the intramyofibrillar glycogen content (or volume relative to fiber volume) is remarkably similar in murine and human muscle (Ortenblad and Nielsen 2015). In contrast, liver glycogen levels are quite similar in the two species (270–400 mmol/kg wet liver) (Pederson et al. 2005b; Testoni et al. 2017; Nilsson and Hultman 1973; Nilsson 1973). The question arises as to why there are such vast differences in muscle glycogen content. Answering this question would require a careful comparison of the rates of glucose metabolism and associated enzyme activities in skeletal muscle, ideally with the same methods. This is difficult to achieve, but some relevant findings are available from the literature. During euglycemic hyperinsulinemia (conditions that favor glycogen synthesis), the rate of muscle glucose uptake in mice ranges from 300 to 400 µmol/min/kg wet wt. Under these conditions, the amount of glucose diverted to glycogen synthesis ranges from ~ 1 to 5% of total glucose uptake, while glycolysis accounts for ≥ 95% of total uptake (Yang et al. 2001; Cha et al. 2011; Fujimoto et al. 2021). In humans, muscle glucose uptake during euglycemic hyperinsulinemia (assuming for simplicity that all of leg weight is accounted for by muscle—assumption made by author) amounts to ~ 60 µmol/min/kg wet wt (basal value ~ 10) (DeFronzo et al. 1985). However, glucose storage (glycogen synthesis) accounts for 65–75% of glucose uptake, whereas glycolysis accounts for the remainder (Young et al. 1988; Christopher et al. 1998). The latter estimates depend on the assumption that whole body flux rates are proportional to what occurs in skeletal muscle, which accounts for ~ 90% of glucose disposal during euglycemic hyperinsulinemia (DeFronzo et al. 1985). Thus, a major reason for the low glycogen content in mouse muscle is that most of the glucose is diverted to glycolysis, likely because of the high metabolic rate of the mouse compared with humans (~ 20-fold higher oxygen consumption at rest in mouse) (Gonzalez and Kuwahira 2018). The implication of this observation is that the resting metabolic rate is a major determinant of muscle glycogen storage. Indeed, Henry and Lowry arrived at a similar conclusion almost 35 years ago when explaining the unusually high glycogen content of canine cardiac Purkinje fibers, namely that the low metabolic rate was primarily responsible for the high glycogen levels (Henry and Lowry 1985).

Despite the marked differences in glycogen content and diversion of glucose 6-P to glycolysis in the mouse, is there another mechanism to explain the higher glycogen content in human muscle? There do not appear to be noteworthy differences in glucose 6-P concentrations in mouse and human skeletal muscle (Blackwood et al. 2021; Blackwood and Katz 2019; Spencer et al. 1992; Katz 1997; Manchester et al. 1996; Ivy et al. 1987). One variable that is not often discussed is the concentration of the substrate for GS, UDP-glucose. The concentration of UDP-glucose in mouse skeletal muscle is ~ 0.05 mmol/kg dry wt (Reynolds et al. 2005; Manchester et al. 1996), and in rat values of ~ 0.15 have been reported, with corresponding glycogen levels of 150–200 mmol/kg dry wt (Piras and Staneloni 1969; Reynolds et al. 2005; Chasiotis 1985). In humans, we consistently measure values of 0.6–1 mmol/kg dry wt (Castillo et al. 1991; Katz et al. 1991a; Raz et al. 1991; Yan et al. 1993). This 10–20-fold higher UDP-glucose content (compared with mouse muscle) corresponds to a concentration of ~ 30 µM, which is below the K_m_ of all forms of GS regardless of phosphorylation state (Roach et al. 1976), suggesting that GS will be much more active in human than in mouse muscle. The reason for the markedly higher UDP-glucose content in human muscle is not clear but may derive from the lower rate of glycolysis (see above). This explanation can be evaluated by studying muscle with a block in glycolysis, as for example in GSD VII (lack of phosphofructokinase activity). Indeed, in two patients afflicted with GSD VII, it was found that the content of UDP-glucose was elevated ~ fivefold and this was associated with a moderate increase in muscle glycogen content (460 and 670 mmol glucosyl units/kg dry wt) (Katz et al. 1991c). However, GS fractional activity was low in the patients, suggesting decreased enzyme activity, which may also have contributed to the increase in UDP-glucose (Katz et al. 1991c). Consistent with this interpretation is the finding that inactivation (phosphorylation) of GS by intense isometric contraction or adrenaline infusion is associated with increased UDP-glucose levels (Raz et al. 1991; Katz and Raz 1995), whereas chronic activation of GS results in decreased UDP-glucose levels (Manchester et al. 1996). It is interesting that in the GSD VII patients all the hexose 6-P contents (including glucose 1-P) were elevated ~ tenfold, suggesting that the large increase in UDP-glucose was primarily a consequence of the inhibition of glycolysis. One might expect that in the presence of such high UDP-glucose and glucose 6-P levels, which should result in near maximal activity of GS (Larner and Villar-Palasi 1971; Roach et al. 1976), glycogen content would be even higher (e.g., McArdle’s patients, GSD V, typically have values that exceed 800 mmol glucosyl units/kg dry wt) (Nielsen et al. 2002). Perhaps, the substantially higher activity of phosphorylase **a** in the GSD VII patients (Katz et al. 1991c) contributed to the lower than expected glycogen values (see above). Noteworthy is that glucose 6-P contents in mouse and human muscle are similar (see above), which is not consistent with the idea that the elevated UDP-glucose content in human muscle derives from elevations in glucose 6-P. Thus, if lower rates of glycolysis are responsible for the marked increase in UDP-glucose in human muscle, it is not clear how the signal is transduced for this to occur.

## Conclusions

**Fig. 4:**
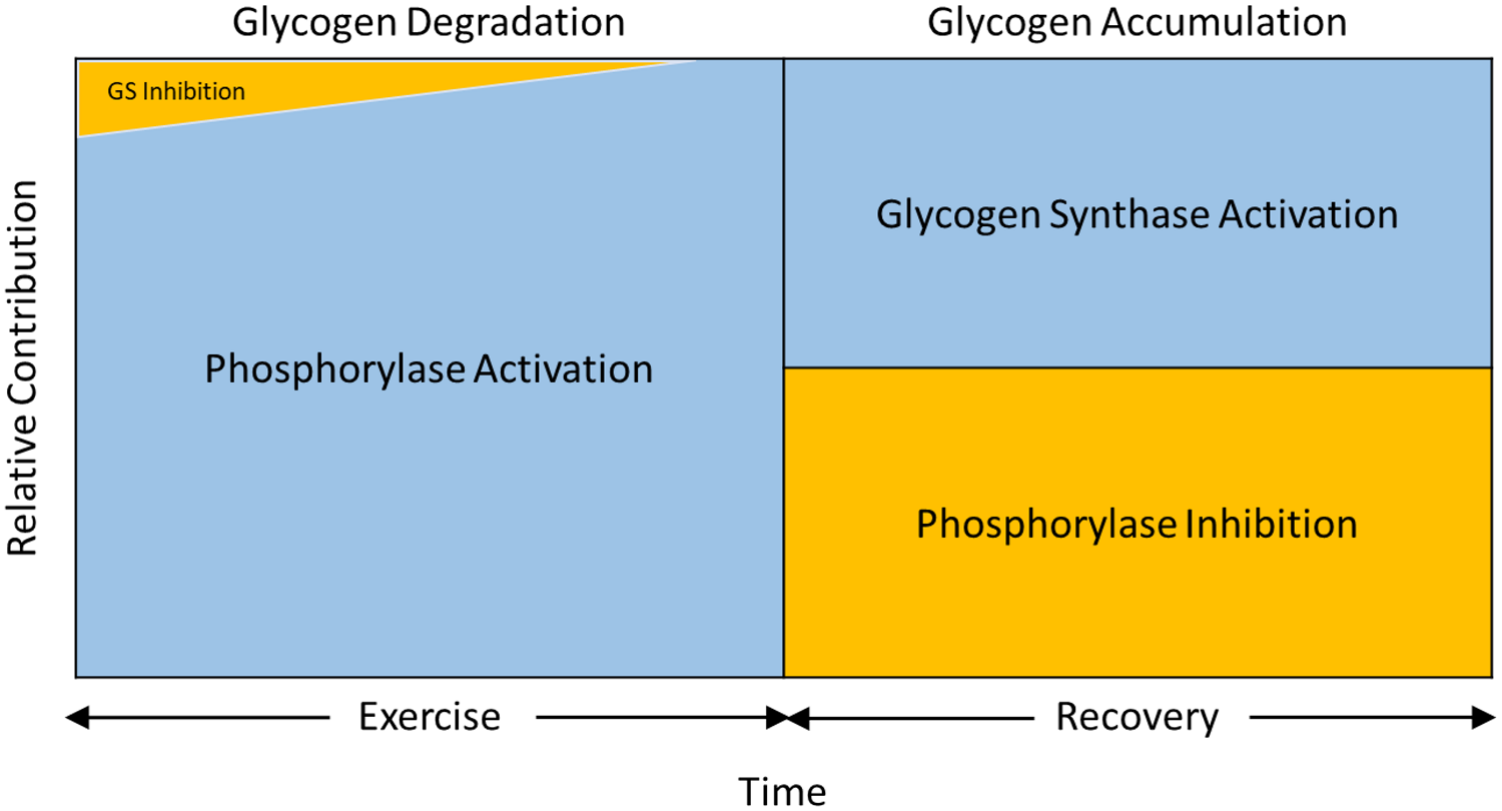
Schematic diagram of glycogen metabolism during exercise and recovery. The relative contributions of enzyme activation or inhibition to glycogen degradation or accumulation are not drawn to scale. Rather the intention is that phosphorylase activation is by far the most important factor for glycogen degradation during exercise, whereas both activation of glycogen synthase (GS) and inhibition of phosphorylase are important components in glycogen accumulation during recovery. See text for additional details

In this review, current views have been presented on the regulation of glycogen metabolism in skeletal muscle with a focus on what occurs during exercise as well as recovery. Based on the data and interpretations presented in this review, a schematic diagram of the regulation of glycogen metabolism during exercise and recovery is presented (Fig. 4). Phosphorylase is one of the most studied proteins in history and considerable information has been accrued on the regulation of the purified enzyme since its discovery. High rates of glycogenolysis are closely linked to the contraction process. Two potential candidates to explain this link are increases in myoplasmic Ca^2+^ and putative AMP transients at the enzymatic site during contraction. However, there is no direct experimental evidence to demonstrate that either of these mechanisms account for activation of phosphorylase and high rates of glycogenolysis in living muscle. Glycogenin was originally believed to be the self-glucosylating protein responsible for initiation of glycogen biogenesis. Recent studies have, however, demonstrated that glycogen synthesis can occur in the absence of functional glycogenin thereby questioning its role in muscle. Activation of glycogen synthase has been viewed as being primarily responsible for glycogen storage after exercise, but recent findings suggest that inactivation of phosphorylase plays a quantitatively significant role in this process as well. Finally, skeletal muscle glycogen levels differ markedly between mice and humans, whereas liver glycogen levels are similar. Muscle glycogen appears to serve different functions in the two species. Thus, despite the plethora of information that has accumulated regarding the regulation of the purified forms of the enzymes of glycogen metabolism, much remains to be elucidated regarding their regulation in living muscle.
